# Associations of dietary intakes of vitamins B_1_ and B_3_ with risk of mortality from CVD among Japanese men and women: the Japan Collaborative Cohort study

**DOI:** 10.1017/S0007114522001209

**Published:** 2023-04-14

**Authors:** Chengyao Tang, Ehab Salah Eshak, Kokoro Shirai, Akiko Tamakoshi, Hiroyasu Iso

**Affiliations:** 1 Public Health, Department of Social Medicine, Graduate School of Medicine, Osaka University, Osaka, Japan; 2 Public Health and Community Medicine Department, Faculty of Medicine, Minia University, Minia, Egypt; 3 Advanced Clinical Epidemiology, Medical Data Science, Public Health, Graduate School of Medicine, Osaka University, Osaka, Japan; 4 Public Health, Department of Social Medicine, Faculty of Medicine, Hokkaido University, Hokkaido, Japan

**Keywords:** Dietary vitamin B_1_, Dietary vitamin B_3_, CVD, Cohort study

## Abstract

The evidence on the association between B vitamins and the risk of CVD is inconclusive. We aimed to examine the association of dietary vitamins B_1_ and B_3_ intakes with risk of CVD mortality among 58 302 Japanese men and women aged 40-79 years participated in the Japan Collaborative Cohort (JACC) study. The Cox proportional hazard model estimated the hazard ratios (HR) and 95% CI of CVD mortality across increasing energy-adjusted quintiles of dietary vitamins B_1_ and B_3_ intakes. During 960 225 person-years of follow-up, we documented a total of 3371 CVD deaths. After adjustment for age, sex, and other CVD risk factors, HR of mortality from ischemic heart disease, myocardial infarction, and heart failure in the highest v. lowest vitamin B_1_ intake quintiles were 0.57 (95 % CI 0·40, 0·80; P_for trend_ < 0·01), 0.56 (95 % CI 0·37, 0·82; P_for trend_ < 0·01), and 0.65 (95 % CI 0·45, 0·96; P_for trend_ = 0·13). The multivariable HR of myocardial infarction mortality in the highest v. lowest vitamin B_3_ intake quintiles was 0.66 (95 % CI 0·48, 0·90; P_for trend_ = 0·02). Atendency towards a reduced risk of haemorrhagic stroke mortality was observed with a higher dietary intake of vitamin B_3_ (HR: 0·74 (95 % CI 0·55, 1·01)) but not vitamin B_1_. In conclusion, higher dietary intakes of vitamins B_1_ and B_3_ were inversely associated with mortality from ischemic heart disease and a higher dietary intake of vitamin B_1_ was inversely associated with a reduced risk of mortality from heart failure among Japanese men and women.

Vitamin B complex exerts its function in energy metabolism, immune function and DNA synthesis, methylation and repair^(1)^. Vitamin B deficiency has been associated with cardiovascular disorders, particularly in ageing population^(1)^.

Among the B complex group, vitamin B_1_ and vitamin B_3_ come mainly from cereals, beef and pork, seeds and nuts, and yeast^(2)^. Vitamin B_1_ deficiency results in beriberi, a neurological and cardiovascular disorder^(3)^, while deficient vitamin B_3_ can cause pellagra-induced dilated cardiomyopathy^(4)^. The potential positive impacts of vitamin B_1_ and B_3_ on cardiovascular health were suggested in several animal studies and supplemental clinical trials. In a Langendorff perfused rat hearts, vitamin B_1_ excreted protective effects against myocardial ischaemic injury via maintaining mitochondrial size and ATP levels^(5)^. A randomised controlled trial on chronic heart failure patients who used diuretics reported that 300 mg/d of vitamin B_1_ supplementation for 28 d increased the left ventricular ejection fraction by 3·9 %^(6)^. On the other hand, 1500–2000 mg/d vitamin B_3_ supplementation was shown to decrease LDL-cholesterol, TAG and lipoprotein(a) levels, while increasing HDL-cholesterol level^(7,8)^. A meta-analysis of clinical trials suggested that vitamin B_3_ supplements significantly reduced major coronary events, stroke and other cardiovascular events^(9)^.

Despite the abundant evidence on cardiovascular beneficial effects of vitamins B_1_ and B_3_ from animal studies^(3,10,11)^ and supplemental clinical trials^(12,13)^, no human observational studies so far have investigated the associations of dietary vitamins B_1_ and B_3_ intakes with risk of CVD. Previous studies indicated that food sources rich in vitamins B_1_ and B_3_ such as fish/seafood and vegetables were associated with a reduced risk of mortality from CVD^(14,15)^. Yet, the evidence on dietary vitamin B complex/CVD association was mainly directed to vitamins B_2_, B_6_, B_12_ and folate, while the effects of dietary vitamins B_1_ and B_3_ intakes were not studied. Another issue is that most of the clinical trials on cardiovascular risk used high dosages of vitamin B_1_ (200–300 mg/d) and B_3_ supplement (1500–2000 mg/d), while the RDA of vitamin B_1_ for Japanese men and women aged 50–69 years were 1·3 and 1·1 mg/d, respectively, and RDA of vitamin B_3_ were 14 and 11 mg/d, respectively^(16)^. Among Japanese men aged 30–49 years, the estimated average requirement and RDA were 1·2 and 1·4 mg/d, respectively, for vitamin B_1_, and those for vitamin B_3_ were 13 and 15 mg/d, respectively. Among Japanese women aged 30–49 years, the corresponding estimated average requirement was 0·9 and 1·1 mg/d, and RDA was 10 and 12 mg/d. The estimated average requirement and RDA of vitamins B_1_ and B_3_ in Japanese men and women aged over 70 years were even less than any other age groups^(16)^. Not all individuals prefer or can afford vitamin supplements for their health; thus, improving the dietary vitamins intakes is more achievable and acceptable by the general population. Therefore, after established effects of supplementary intakes of vitamins B_1_ and B_3_ have been determined, studying the cardiovascular impacts of dietary intakes of vitamins B_1_ and B_3_ is now warranted. Owing to the research gap in the field of epidemiology and similar food sources of vitamins B_1_ and B_3_, their associations with CVD mortality were hypothesised in the present study. Therefore, we aimed to investigate the associations of dietary vitamins B_1_ and B_3_ intakes with risk of CVD mortality, which was considered as a proxy of CVD incidence risk, among Japanese men and women using the Japan Collaborative Cohort (JACC) study, a nationwide, community-based prospective cohort study.

## Methods

### Study population and baseline data

Under the sponsorship of the Ministry of Education, Sports, and Science, the JACC study had the baseline survey (1988–1990) of 110 585 Japanese men (*n* 46 395) and women (*n* 64 190) aged 40–79 years from forty-five areas all over Japan. A detailed cohort profile of the JACC study was published previously^(17)^. Data on the baseline lifestyle and participants’ characteristics, including demographic data, medical history of chronic diseases, diabetes mellitus, hypertension, smoking, alcohol consumption, exercise, diet and other items, were compiled via a self-administered questionnaire (online Supplementary Methods). The questionnaire included a validated forty-food item/FFQ which was distributed in thirty-two areas; therefore, we started with 86 401 subjects from those thirty-two areas. After the exclusion of non-respondents to FFQ (*n* 24 614), we further excluded those who reported a medical history of CVD or cancer (*n* 3142) and those who had implausible energy intakes defined as outliers of mean ± three standard deviations (*n* 343). Finally, a total of 58 302 individuals were eligible for the present study (22 989 men and 35 313 women) (online Supplementary Fig. S1). Written informed consent was acquired from community leaders or the individuals. The protocol of JACC study was approved by the Medical Ethical Committees of Nagoya University School of Medicine.

### Dietary intake assessment

The participants were required to choose one from five frequency responses to describe the usual consumption frequency of forty food and beverage items over the past 12 months without specification of the portion size. The five responses were rarely, 1–2 times/month, 1–2 times/week, 3–4 times/week and almost every day. These frequencies were transformed into weekly consumption scores of 0, 0·38, 1·5, 3·5 and 7·0 per week, respectively^(17,18)^. A validation study among eighty-five individuals using four 3-d weighed dietary records over a 1-year period as a reference standard determined the portion size for each food and validated the FFQ intakes. The amount of nutrients in each food was calculated by multiplying the weekly consumption scores by the estimated portion size. The values of vitamins B_1_ and B_3_ and other nutrients from each food category were calculated according to the Standardised Tables of Food Composition, 5th revised version^(19)^ which listed the nutrients content in 100 mg of different foods. Thus, the total vitamins B_1_ and B_3_ intakes were calculated by summing their intakes from all over the foods in the FFQ. The details of computation of nutrient intakes from FFQ^(18)^ and the accuracy of food composition tables in Japan^(20,21)^ were published previously. The Spearman rank correlation coefficients for vitamins B_1_ and B_3_ intakes between the FFQ and the four 3-d dietary records were 0·36 and 0·32, respectively, after energy adjustment^(22)^. The energy-adjusted mean ± standard deviation intakes in mg/d from weighed dietary record and FFQ were 1·08 (sd 0·20) and 0·71 (sd 0·20) for vitamin B_1_, but the respective values for vitamin B_3_ were not reported^(18)^.

### Mortality surveillance

The investigators annually or biannually confirmed the dates and causes of death in each area^(17)^. The International Classification of Diseases, 10th revision (ICD10) codes were applied to determine the underlying causes of death. In this study, our primary outcome was the total CVD mortality (ICD I01-I99). Cause-specific outcomes included mortalities from total stroke (ICD I60-I69), haemorrhagic stroke (ICD I60-I61), ischaemic stroke (ICD I63.0-I63.9), ischaemic heart disease (ICD I20-I25), myocardial infarction (ICD I20) and heart failure (ICD I50). This death certificate ascertainment was applied to all deaths within our cohort except for deaths that occurred outside of the original resident areas, which were treated as censored cases.

### Statistical analysis

Energy-adjusted dietary intakes of vitamins B_1_ and B_3_ were categorised into five categorical groups (quintiles). The significance of differences in means or proportions of participants’ characteristics and known risk factors of CVD in each quintile was tested by the ANCOVA and *χ* ^2^ test.

Person-years of follow-up were calculated from the baseline in 1988–1990 to their first endpoint in this follow-up as follows: death, moving out or the end of follow-up, whichever came first. The follow-up for mortality from CVD was conducted until 31 December 2009 in general; however, in four areas the follow-up was stopped until 31 December 1999, in another four areas until 31 December 2003 and in two areas until 31 December 2008^(17)^. The Cox proportional hazard model was applied to calculate crude and multivariable-adjusted hazard ratios and 95 % CI for risk of mortality from CVD during the follow-up period (1988–2009) across quintiles of dietary vitamins B_1_ and B_3_ intakes. We confirmed no violation of the Cox proportional hazard assumption because there were no significant interactions between the categorical rank variables of dietary vitamins B_1_ and B_3_ intakes and a function of survival time for all the tested outcomes. Multiplicative interactions of vitamins B_1_ and B_3_ with sex were tested to decide on presenting the data sex specifically or for combined men and women. The hypothesised confounders included age, sex, medical history of hypertension and diabetes, smoking status, ethanol intake, hours of sports, hours of walking, quintiles of BMI, years of education, perceived mental stress, daily utilisation of multivitamin supplementation, energy-adjusted quintiles of Na and SFA intakes and quintiles of total energy intake. Details of these factors are given in online Supplementary Methods.

We assigned the median values to each quintile of vitamins B_1_ and B_3_ and tested their significance to calculate the trends across quintiles of vitamins B_1_ and B_3_ intakes. We further conducted a sensitivity analysis by excluding those who died within first 3 years of follow-up to avoid potential as-yet-undiagnosed diseases at baseline. All probability values for statistical test were two-tailed, and *P* < 0·05 was regarded as statistically significant. We applied the SAS statistical package (Version 9.4; SAS Institute Inc.) for statistical analysis.

## Results

As shown in Table 1, participants in the highest quintile of both vitamins B_1_ and B_3_ intake were older, less educated, under less mental stress, had more walking time, had higher BMI and were less likely to be current smoker and to have a history of hypertension or diabetes. They also used multivitamin supplementation less frequently and consumed less alcohol but consumed more Na, SFA and total energy when compared with those in the lowest quintile. In this study, sources of vitamin B_1_ were 31 % from pork, 17 % from vegetables, 10 % from fish and 7 % from potatoes, while sources of vitamin B_3_ were 43 % from fish, 13 % from vegetables, 8 % from pork, 7 % from coffee and 6 % from green tea (data not shown in tables).

**Table 1.** Participants’ characteristics and dietary variables according to quintiles of dietary vitamins B_1_ and B_3_ intakes at baseline in a cohort of 22 989 men and 35 313 women with a total of 3371 CVD mortality cases

Quintile	1 (low)	2	3	4	5 (high)	*P* for difference
No. at risk	11 660	11 661	11 660	11 661	11 660	
Vitamin B_1_						
Vitamin B_1_ intake, mg/d	0·8	0·9	1·0	1·0	1·2	< 0·001
Female, %	21·1	57·2	69·9	76·3	78·3	< 0·001
Age, years	54·8	56·3	56·3	56·6	56·8	< 0·001
BMI, kg/m^2^	22·7	22·8	22·8	23·0	22·9	< 0·001
Ethanol intake, g/d	42·8	21·5	17·3	14·1	12·8	< 0·001
Current smoker, %	52·4	26·8	18·1	14·1	12·7	< 0·001
History of hypertension, %	20·8	20·7	20·2	19·8	18·4	< 0·001
History of diabetes, %	5·6	4·9	4·5	4·2	3·5	< 0·001
Sports ≥ 5 h/week, %	5·6	5·3	5·1	5·2	5·7	0·14
Walking ≥ 1 h/day, %	47·7	48·4	49·2	52·1	56·4	< 0·001
> 18 years education, %	16·7	13·9	13·6	12·9	11·8	< 0·001
High mental stress, %	26·2	23·3	21.0	20·9	20·2	< 0·001
Multivitamin supplementation, %	3·9	3·4	3·0	3·2	3·0	< 0·001
Na intake, mg/d	1498·4	1779·6	2005·3	2221·4	2515·4	< 0·001
SFA intake, mg/d	8·0	9·2	9·8	10·4	11·9	< 0·001
Total energy intake, kcal/d	1665·6	1446·3	1457·5	1512·2	1680·2	< 0·001
Vitamin B_3_ intake, mg/d	15·2	17·3	18·1	18·9	20·4	< 0·001
						
Vitamin B_3_						
Vitamin B_3_ intake, mg/d	14·4	16·7	17·9	19·2	21·5	< 0·001
Female, %	30·1	59·6	67·4	71·3	74·4	< 0·001
Age, years	55·7	56·2	56·1	56·2	56·7	< 0·001
BMI, kg/m^2^	22·7	22·8	22·8	22·9	23·0	< 0·001
Ethanol intake, g/d	42·3	23·8	19·7	16·9	13·5	< 0·001
Current smoker, %	42·9	24·5	20·7	18·8	17·6	< 0·001
History of hypertension, %	22·0	20·8	19·6	19·5	18·0	< 0·001
History of diabetes, %	5·8	4·6	4·1	4·1	4·1	< 0·001
Sports ≥ 5 h/week, %	5·8	4·7	5·2	5·5	5·7	0·88
Walking ≥ 1 h/day, %	47·5	47·7	50·5	52·0	56·0	< 0·001
> 18 years education, %	17·5	14·2	13·1	12·6	11·5	< 0·001
High mental stress, %	24·8	23·1	22·2	21·0	20·5	< 0·001
Multivitamin supplementation, %	4·2	3·6	2·9	3·0	3·0	< 0·001
Na intake, mg/d	1678·5	1894·9	2016·8	2113·8	2316·1	< 0·001
SFA intake, mg/d	8·8	9·6	9·8	10·1	10·9	< 0·001
Total energy intake, kcal/d	1639·1	1461·6	1478·0	1536·6	1646·6	< 0·001
Vitamin B_1_ intake, mg/d	0·8	0·9	1·0	1·0	1·1	< 0·001

Since no interaction with sex was observed for the association of vitamins B_1_ and B_3_ with CVD and specific endpoints, we combined the results of men and women in the main analyses. During 960 225 person-years of follow-up for 58 302 participants, we documented a total of 3371 deaths due to CVD, among whom there were 1504 deaths due to stroke (549 of which were due to haemorrhagic stroke and 816 of which were due to ischaemic stroke), 699 deaths were due to ischaemic heart disease (including 524 deaths due to myocardial infarction) and 564 deaths were due to heart failure.

As shown in Table 2, the dietary intake of vitamin B_1_ was not associated with mortality from total stroke or its subtypes. On the other hand, a higher dietary vitamin B_1_ intake was associated with the reduced risk of ischaemic heart disease, myocardial infarction and total CVD; hazard ratios were 0·57 (95 % CI 0·40, 0·80; *P*_for trend_ < 0·01), 0·56 (95 % CI 0·37, 0·82; *P*_for trend_ < 0·01) and 0·85 (95 % CI 0·73, 0·99; *P*_for trend_ = 0·03), respectively, in the highest *v*. lowest intake quintile. Moreover, the multivariable-adjusted hazard ratio of heart failure mortality in the highest *v*. lowest intake quintiles was 0·65 (95 % CI 0·45, 0·96; *P*_for trend_ = 0·13).

**Table 2.** CVD mortality according to quintiles of vitamin B_1_ intake (Hazard ratios and 95 % confidence intervals)

			Q2		Q3		Q4		Q5 (high)		
		Q1 (low)	HR	95 % CI	HR	95 % CI	HR	95 % CI	HR	95 % CI	*P*_for trend_
Total *n*		11 660	11 661		11 660		11 661		11 660		
Person-years		182 559	185 652		192 066		197 635		202 313		
CVD											
	No. of case	675	701		651		674		670		
	Age- and sex-adjusted HR (95% CI)	1·00	0·92	0·83, 1·03	0·86	0·77, 0·96	0·83	0·75, 0·93	0·80	0·71, 0·89	< 0·001
	Multivariable HR (95% CI)^1^	1·00	0·92	0·83, 1·03	0·86	0·77, 0·96	0·84	0·75, 0·93	0·80	0·71, 0·89	< 0·001
	Multivariable HR (95% CI)^2^	1·00	1·01	0·90, 1·14	0·93	0·82, 1·05	0·90	0·79, 1·02	0·86	0·75, 0·98	0·01
	Multivariable HR (95% CI)^3^	1·00	0·98	0·87, 1·11	0·88	0·76, 1·00	0·85	0·74, 0·98	0·85	0·73, 0·99	0·03
Stroke											
	No. of case	284	300		290		313		317		
	Age- and sex-adjusted HR (95% CI)	1·00	0·94	0·80, 1·11	0·91	0·77, 1·08	0·93	0·78, 1·10	0·91	0·76, 1·07	0·27
	Multivariable HR (95% CI)^1^	1·00	0·94	0·79, 1·11	0·92	0·77, 1·09	0·93	0·78, 1·10	0·90	0·76, 1·07	0·28
	Multivariable HR (95% CI)^2^	1·00	1·11	0·92, 1·33	1·08	0·89, 1·31	1·10	0·90, 1·34	1·07	0·87, 1·30	0·68
	Multivariable HR (95% CI)^3^	1·00	1·06	0·87, 1·28	1·00	0·81, 1·23	1·02	0·82, 1·27	1·05	0·83, 1·33	0·77
Haemorrhagic stroke											
	No. of case	119	105		101		104		120		
	Age- and sex-adjusted HR (95% CI)	1·00	0·79	0·60, 1·04	0·74	0·56, 0·97	0·71	0·54, 0·95	0·79	0·60, 1·04	0·09
	Multivariable HR (95% CI)^1^	1·00	0·79	0·60, 1·04	0·74	0·56, 0·98	0·73	0·55, 0·96	0·80	0·61, 1·05	0·15
	Multivariable HR (95% CI)^2^	1·00	0·94	0·70, 1·27	0·89	0·65, 1·23	0·88	0·64, 1·22	0·97	0·70, 1·33	0·93
	Multivariable HR (95% CI)^3^	1·00	0·91	0·66, 1·24	0·85	0·61, 1·19	0·84	0·59, 1·21	1·02	0·70, 1·50	0·74
Ischaemic stroke											
	No. of case	141	169		161		175		170		
	Age- and sex-adjusted HR (95% CI)	1·00	1·05	0·84, 1·32	1·03	0·82, 1·30	1·04	0·83, 1·31	0·99	0·79, 1·25	0·89
	Multivariable HR (95% CI)^1^	1·00	1·04	0·83, 1·31	1·03	0·81, 1·30	1·03	0·82, 1·30	0·98	0·77, 1·23	0·83
	Multivariable HR (95% CI)^2^	1·00	1·22	0·95, 1·57	1·18	0·90, 1·54	1·18	0·90, 1·54	1·12	0·85, 1·48	0·62
	Multivariable HR (95% CI)^3^	1·00	1·13	0·87, 1·47	1·06	0·80, 1·41	1·08	0·80, 1·45	1·06	0·77, 1·47	0·99
Ischaemic heart disease											
	No. of case	174	163		134		122		106		
	Age- and sex-adjusted HR (95% CI)	1·00	0·90	0·72, 1·11	0·75	0·60, 0·95	0·65	0·51, 0·83	0·55	0·43, 0·71	< 0·001
	Multivariable HR (95% CI)^1^	1·00	0·89	0·72, 1·11	0·75	0·60, 0·95	0·65	0·51, 0·83	0·55	0·42, 0·71	< 0·001
	Multivariable HR (95% CI)^2^	1·00	0·90	0·71, 1·15	0·74	0·57, 0·96	0·64	0·48, 0·84	0·54	0·40, 0·71	< 0·001
	Multivariable HR (95% CI)^3^	1·00	0·89	0·69, 1·14	0·73	0·55, 0·96	0·64	0·47, 0·87	0·57	0·40, 0·80	< 0·001
Myocardial infarction											
	No. of case	133	122		101		90		78		
	Age- and sex-adjusted HR (95% CI)	1·00	0·90	0·70, 1·15	0·77	0·58, 1·00	0·65	0·49, 0·87	0·55	0·41, 0·74	< 0·001
	Multivariable HR (95% CI)^1^	1·00	0·89	0·69, 1·15	0·76	0·58, 1·00	0·65	0·49, 0·86	0·54	0·40, 0·73	< 0·001
	Multivariable HR (95% CI)^2^	1·00	0·90	0·68, 1·19	0·74	0·55, 1·01	0·63	0·46, 0·87	0·53	0·38, 0·74	< 0·001
	Multivariable HR (95% CI)^3^	1·00	0·88	0·66, 1·17	0·73	0·53, 1·00	0·63	0·44, 0·90	0·56	0·37, 0·82	0·001
Heart failure											
	No. of case	103	118		107		117		119		
	Age- and sex-adjusted HR (95% CI)	1·00	0·91	0·69, 1·19	0·80	0·60, 1·06	0·80	0·60, 1·05	0·78	0·59, 1·03	0·06
	Multivariable HR (95% CI)^1^	1·00	0·90	0·69, 1·18	0·80	0·60, 1·06	0·81	0·61, 1·07	0·79	0·60, 1·04	0·34
	Multivariable HR (95% CI)^2^	1·00	0·91	0·67, 1·23	0·77	0·56, 1·06	0·78	0·57, 1·07	0·75	0·55, 1·04	0·27
	Multivariable HR (95% CI)^3^	1·00	0·87	0·64, 1·18	0·69	0·49, 0·97	0·67	0·47, 0·96	0·65	0·45, 0·96	0·13

^1^Adjusted for age, sex, and socio-economic status (educational status).

^2^Adjusted for age, sex, socio-economic status (educational status), and health behaviours (hours of walking, hours of sports, ethanol intake and smoking status).

^3^A full mode with adjustment for age, sex, educational status, hours of walking, hours of sports, ethanol intake, smoking status, history of hypertension and diabetes, BMI, perceived mental stress, multivitamin supplementation, quintiles of energy-adjusted Na and SFA intakes and total energy intakes.

For vitamin B_3_, as shown in Table 3, there was no association with the mortality from stroke or heart failure. Statistically significant inverse trends in risks of mortality from total CVD, haemorrhagic stroke, ischaemic heart disease and myocardial infarction were observed in the age- and sex-adjusted model. However, after the multivariate adjustment, these associations were weakened; the multivariable-adjusted hazard ratios in the highest *v*. lowest quintiles of dietary vitamin B_3_ were 0·90 (95 % CI 0·80, 1·03; *P*_for trend_ = 0·13) for total CVD mortality, 0·74 (95 % CI 0·55, 1·01; *P*_for trend_ = 0·16) for haemorrhagic stroke, 0·79 (95 % CI 0·60, 1·04; *P*_for trend_ = 0·05) for ischaemic heart disease and 0·66 (95 % CI 0·48, 0·90; *P*_for trend_ = 0·02) for myocardial infarction.

**Table 3.** CVD mortality according to quintiles of vitamin B_3_ intake (Hazard ratios and 95 % confidence intervals)

			Q2		Q3		Q4		Q5 (high)		
		Q1 (low)	HR	95 % CI	HR	95 % CI	HR	95 % CI	HR	95 % CI	*P*_for trend_
Total *n*		11 660	11 661		11 660		11 661		11 660		
Person-years		180 265	187 575		194 533		197 250		200 601		
CVD											
	No. of case	715	658		663		649		686		
	Age- and sex-adjusted HR (95% CI)	1·00	0·89	0·80, 0·99	0·88	0·79, 0·98	0·85	0·76, 0·94	0·86	0·77, 0·95	0·004
	Multivariable HR (95% CI)^1^	1·00	0·89	0·80, 0·99	0·88	0·79, 0·98	0·84	0·75, 0·94	0·85	0·77, 0·95	0·004
	Multivariable HR (95% CI)^2^	1·00	0·93	0·83, 1·05	0·92	0·82, 1·03	0·87	0·78, 0·99	0·88	0·78, 1·00	0·03
	Multivariable HR (95% CI)^3^	1·00	0·92	0·82, 1·04	0·91	0·81, 1·03	0·88	0·78, 0·99	0·90	0·80, 1·03	0·13
Stroke											
	No. of case	316	268		309		311		300		
	Age- and sex-adjusted HR (95% CI)	1·00	0·82	0·69, 0·97	0·93	0·79, 1·09	0·92	0·78, 1·08	0·85	0·72, 1·00	0·17
	Multivariable HR (95% CI)^1^	1·00	0·81	0·69, 0·96	0·92	0·78, 1·08	0·91	0·77, 1·07	0·84	0·71, 0·99	0·08
	Multivariable HR (95% CI)^2^	1·00	0·90	0·75, 1·07	1·02	0·85, 1·21	1·00	0·84, 1·19	0·93	0·77, 1·11	0·46
	Multivariable HR (95% CI)^3^	1·00	0·87	0·73, 1·04	0·98	0·82, 1·17	0·97	0·80, 1·16	0·91	0·75, 1·10	0·38
Haemorrhagic stroke											
	No. of case	138	94		104		101		112		
	Age- and sex-adjusted HR (95% CI)	1·00	0·63	0·49, 0·83	0·68	0·52, 0·88	0·64	0·49, 0·83	0·68	0·52, 0·88	0·009
	Multivariable HR (95% CI)^1^	1·00	0·63	0·49, 0·83	0·67	0·52, 0·88	0·64	0·49, 0·83	0·67	0·52, 0·88	0·01
	Multivariable HR (95% CI)^2^	1·00	0·69	0·52, 0·91	0·74	0·55, 0·98	0·69	0·52, 0·93	0·73	0·55, 0·98	0·13
	Multivariable HR (95% CI)^3^	1·00	0·67	0·50, 0·89	0·71	0·53, 0·95	0·68	0·50, 0·92	0·74	0·55, 1·01	0·16
Ischaemic stroke											
	No. of case	155	139		179		182		162		
	Age- and sex-adjusted HR (95% CI)	1·00	0·87	0·69, 1·10	1·13	0·91, 1·41	1·12	0·90, 1·40	0·96	0·77, 1·21	0·67
	Multivariable HR (95% CI)^1^	1·00	0·86	0·68, 1·09	1·11	0·89, 1·38	1·11	0·89, 1·38	0·95	0·76, 1·20	0·98
	Multivariable HR (95% CI)^2^	1·00	0·95	0·75, 1·22	1·23	0·97, 1·56	1·21	0·95, 1·55	1·05	0·81, 1·35	0·69
	Multivariable HR (95% CI)^3^	1·00	0·92	0·72, 1·18	1·17	0·92, 1·50	1·17	0·91, 1·50	1·01	0·77, 1·32	0·96
Ischaemic heart disease											
	No. of case	174	149		136		115		125		
	Age- and sex-adjusted HR (95% CI)	1·00	0·89	0·71, 1·11	0·81	0·64, 1·02	0·68	0·53, 0·86	0·71	0·56, 0·90	< 0·001
	Multivariable HR (95% CI)^1^	1·00	0·88	0·71, 1·10	0·80	0·64, 1·01	0·67	0·53, 0·86	0·71	0·56, 0·90	0·001
	Multivariable HR (95% CI)^2^	1·00	0·88	0·70, 1·12	0·80	0·62, 1·02	0·66	0·51, 0·86	0·69	0·53, 0·90	0·002
	Multivariable HR (95% CI)^3^	1·00	0·90	0·71, 1·15	0·84	0·65, 1·08	0·72	0·55, 0·95	0·79	0·60, 1·04	0·05
Myocardial infarction											
	No. of case	134	119		95		86		90		
	Age- and sex-adjusted HR (95% CI)	1·00	0·93	0·72, 1·20	0·75	0·57, 0·98	0·67	0·51, 0·89	0·68	0·51, 0·89	< 0·001
	Multivariable HR (95% CI)^1^	1·00	0·93	0·72, 1·19	0·74	0·57, 0·97	0·67	0·50, 0·88	0·68	0·51, 0·89	< 0·001
	Multivariable HR (95% CI)^2^	1·00	0·92	0·70, 1·20	0·72	0·54, 0·96	0·64	0·47, 0·87	0·64	0·48, 0·87	0·001
	Multivariable HR (95% CI)^3^	1·00	0·91	0·70, 1·20	0·72	0·54, 0·97	0·65	0·48, 0·88	0·66	0·48, 0·90	0·02
Heart failure											
	No. of case	103	119		114		99		129		
	Age- and sex-adjusted HR (95% CI)	1·00	1·03	0·79, 1·35	0·96	0·73, 1·27	0·81	0·61, 1·07	1·01	0·77, 1·31	0·60
	Multivariable HR (95% CI)^1^	1·00	1·02	0·78, 1·34	0·95	0·73, 1·25	0·80	0·60, 1·06	1·00	0·76, 1·31	0·81
	Multivariable HR (95% CI)^2^	1·00	1·04	0·78, 1·39	0·95	0·71, 1·28	0·79	0·58, 1·07	0·97	0·72, 1·31	0·97
	Multivariable HR (95% CI)^3^	1·00	1·03	0·77, 1·38	0·95	0·70, 1·28	0·79	0·57, 1·08	0·97	0·71, 1·33	0·88

^1^Adjusted for age, sex and socio-economic status (educational status).

^2^Adjusted for age, sex, socio-economic status (educational status) and health behaviours (hours of walking, hours of sports, ethanol intake and smoking status).

^3^A full mode with adjustment for age, sex, educational status, hours of walking, hours of sports, ethanol intake, smoking status, history of hypertension and diabetes, smoking status, BMI, hours of walking, hours of sports, educational status, perceived mental stress, ethanol intake, multivitamin supplementation, quintiles of energy-adjusted Na and SFA intakes and total energy intakes.

There were 456 participants who died within the first 3 years of follow-up, and excluding those subjects yielded no substantial changes in the associations of vitamins B_1_ and B_3_ with mortality from ischaemic heart disease and myocardial infarction (online Supplementary Table S1).

## Discussion

In this large community-based prospective cohort study of Japanese men and women, higher dietary intakes of vitamins B_1_ and B_3_ were associated with reduced risks of mortality from total CVD, ischaemic heart disease and myocardial infarction. Neither dietary vitamin B_1_ nor vitamin B_3_ intake was associated with the mortality risk of stroke, except for a tendency towards a reduced risk of haemorrhagic stroke with a higher dietary vitamin B_3_ intake. Moreover, a higher dietary intake of vitamin B_1_ was associated with a reduced risk of heart failure.

As far to our knowledge, the present study is the first to investigate associations of dietary vitamins B_1_ and B_3_ intakes with risk of CVD mortality despite the abundant evidence from animal studies and clinical trials on vitamins B_1_ and B_3_ supplements. Vitamins B_1_ and B_3_ in animal studies and human clinical trials showed protective effects against myocardial ischaemia.

One study on dogs showed that administration of vitamin B_1_ decreased the metabolic needs of the heart, which was manifested as reduced myocardial oxygen consumption, mean peripheral pressure and left ventricular pressure up to 45, 25 and 10 % respectively^(11)^. A clinical trial on ten healthy adults and ten type 2 diabetes patients reported improvements in the brachial artery vasoactivity and the endothelium-dependent vasodilatation in both groups after a week of daily intravenous administration of 100 mg of vitamin B_1_^(23)^. Another randomised, cross-over and investigator-blinded trial on twenty adult healthy volunteers indicated the flow-mediated dilatation of the brachial artery was reduced by 50 % of its baseline diameter after smoking one cigarette, and the reduction in the flow-mediated vasodilatation with smoking one cigarette was only 25 % when 1050 mg/d oral benfotiamine was administered for 3 d before the experiment^(24)^.

On the other hand, vitamin B_3_ is a candidate to lower the risk of CVD as it is known to decrease LDL-cholesterol, TAG and lipoprotein(a) levels, while increasing HDL-cholesterol level^(7)^. Among 8341 American men aged 30–64 years from Coronary Drug Project with previous myocardial infarction, 3000 mg/d vitamin B_3_ *v*. placebo for a follow-up of 15 years reduced 14 % of the mortality from total CVD and 26 % of the mortality from ischaemic attack after a mean follow-up of 15 years^(12)^. Additionally, a meta-analysis of twenty-three randomised controlled trials including 39 195 participants reported a pooled risk ratio (CI) of mortality from fatal or non-fatal myocardial infarction (OR: 0·93; 95 % CI 0·87, 1·00) for vitamin B_3_ (median dose: 2 g/d; median duration: 11·5 months) *v*. control^(13)^. Another meta-analysis of eleven randomised controlled trials including 6616 participants showed vitamin B_3_ (250–3000 mg/d) significantly decreased major coronary events (OR: 0·75; 95 % CI 0·65, 0·96), stroke (OR: 0·74; 95 % CI 0·59, 0·92) and any cardiovascular events (OR: 0·73; 95 % CI 0·63, 0·85)^(9)^. In a recent meta-analysis of seventeen clinical trials including 35 760 participants, vitamin B_3_ therapy (100–4000 mg/d) was shown to be associated with reduction of acute coronary syndrome (relative risk: 0·74; 95 % CI 0·58, 0·96) and stroke (relative risk: 0·74; 95 % CI 0·59, 0·94)^(25)^. A meta-analysis including 9959 subjects reported similar results for total CVD events (OR: 0·66; 95 % CI 0·49, 0·89) and major coronary events (OR: 0·75; 95 % CI 0·59, 0·96) but not for stroke (OR: 0·88; 95 % CI 0·50, 1·54)^(26)^. Another meta-analysis of thirteen trials of vitamin B_3_ treatment demonstrated a significant reduced risk of non-fatal myocardial infarction (risk ratio: 0·85; 95 % CI 0·73, 1·00), a weak association with CVD mortality (risk ratio: 0·91; 95 % CI 0·81, 1·02) and no association with stroke (risk ratio: 0·89; 95 % CI: 0·72, 1·10)^(27)^.

The mechanisms by which vitamins B_1_ and B_3_ might be protective against CVD mortality, especially those from ischaemic heart disease could be summarised here. Vitamin B_1_ deficiency was highly prevalent in patients with type 2 diabetes^(28)^, which is considered as one of the risk factors for ischaemic heart disease. In addition, vitamin B_1_ inhibits human infragenicular accelerated proliferation of arterial smooth muscle cells and mitigates atherosclerosis and endothelial dysfunction^(29)^. Another potential mechanism might be the protective effects of vitamin B_1_ against ischaemic injury via reducing the metabolic needs of heart^(5)^. For vitamin B_3_, the reduced CVD risk may be involved in the favourable effects of vitamin B_3_ on lipid metabolism^(7,8)^. Vitamin B_3_ also has anti-inflammatory properties demonstrated by lowering C-reactive protein lipoprotein-associated phospholipase A2, inhibiting pro-atherogenic chemokines and enhancing serum levels of adiponectin^(26)^. Moreover, an antihypertensive effect of vitamin B_3_ was also suggested^(30)^.

We observed that a higher dietary vitamin B_1_ intake was associated with reduced risk of mortality from heart failure. Vitamin B_1_ deficiency was commonly considered to be correlated with a failing heart. In a meta-analysis of nine observational studies, the prevalence of vitamin B_1_ deficiency was higher with an OR of 2·5 (95 % CI 1·7, 3·9) in heart failure group than in control^(31)^. Also known as wet beriberi or cardiac beriberi, vitamin B_1_ deficiency was characterised by peripheral neuropathy and muscle weakness resulting in heart failure^(3)^. The vitamin B_1_ deficiency-related heart failure was attributed to the vitamin B_1_ role in energy metabolism^(3)^. Some studies reported that vitamin B_1_ supplementation had beneficial effects on cardiac function^(6)^, but the evidence is still inconclusive^(3)^.

The dietary intake of vitamin B_3_ in our study tended to associate with a lower risk of mortality from haemorrhagic stroke. In a case–control study including sixty-nine stroke cases and sixty-nine controls, vitamin B_3_ was found to be inversely correlated with risk of stroke (OR: 0·17; 95 % CI 0·04, 0·82)^(32)^. However, some meta-analyses concluded that vitamin B_3_ had similar protective effects on both CHD and stroke outcomes^(9,25)^, while others failed to find protective effects against stroke^(26,27)^. The available studies and meta-analyses did not comment on the effect of vitamin B_3_ on stroke subtypes. Vitamin B_3_ was shown to reduce the blood pressure, an important risk factor for haemorrhagic stroke^(30)^. On the other hand, vitamin B_3_ may promote vascular plasticity after an acute attack of stroke. In an animal experiment, Niaspan treatment of stroke in rats with diabetes promoted vascular remodelling and improved functional outcome^(33)^. Therefore, an impact of vitamin B_3_ on risk of haemorrhagic stroke mortality could be plausible.

To the best of our knowledge, this is the first study to investigate the association of dietary intakes of vitamins B_1_ and B_3_ with the risk of mortality from CVD among Japanese population. The JACC study is a large, nationwide, community-based, prospective Japanese cohort study. The large sample size allowed us to investigate the associations of quintile categories of dietary vitamins B_1_ and B_3_ intakes with the risks of type-specific cardiovascular mortality as well as total CVD in Japanese population. Other strengths of this study included the prospective study design, the utilisation of a validated FFQ, the consistent endpoint determination and the exclusion of participants with CVD and cancer before the starting point of follow-up.

Limitations of this study mainly originate from the dietary assessment. The one-time measurement of dietary intakes cannot completely represent the consumption of nutrients during a long-term follow-up. The exclusion of 18 428 non-respondents to FFQ might result in a selection bias. Compared with 24 614 non-respondents to FFQ, the 61 787 respondents were more likely to be young and more educated (online Supplementary Table S2). Several research of the JACC study reported the underestimation of nutrients intakes, which could be attributed to the limited number of food items in the used FFQ. Second, we had no data about the exact amounts or types of vitamin supplementation. To our knowledge, in the past century, vitamin supplementation was not common among Japanese population; thus, we believe that it would not affect the result substantially. In this study, approximately 88 % of participants did not use any vitamin supplementation and only 3 % used it on daily basis. The exclusion of daily supplementation uses did not change the result. Third, in Japan, the accuracy of heart failure death certificate diagnosis is a questionable issue. It is generally believed that heart failure death was overestimated before 1994, because most deaths of unknown origin such as cardiac arrest or arrhythmic death were more likely diagnosed as unspecific heart failure^(34)^. Therefore, approximately 27–50 % heart failure deaths were accounted for this misclassification^(34)^. Lastly, we did not have data on biomarkers of atherosclerosis and endothelial dysfunction, lipid metabolism and systematic inflammation such as C-reactive protein, lipoprotein-associated phospholipase A2, pro-atherogenic chemokines or serum levels of adiponectin and cannot determine all confounding effects from some other nutrients, lifestyles and socio-economic factors.

### Conclusions

In this prospective cohort study, higher dietary intakes of vitamins B_1_ and B_3_ were inversely associated with a reduced risk of mortality from CVD among Japanese men and women. Dietary intakes of these vitamins from their food sources are available, accessible, affordable, safe and more acceptable by the general population than supplementary intakes. Therefore, dietary intakes of food rich in these vitamins could be encouraged for decreasing the risk of CVD mortality. However, our findings warrant further studies in different populations.

## Supplementary material

For supplementary material accompanying this paper visit https://doi.org/10.1017/S0007114522001209. click here to view supplementary material
