# Supplementary material for: Associations of dietary intakes of vitamins B1 and B3 with risk of mortality from CVD among Japanese men and women: the Japan Collaborative Cohort study
Source: Br J Nutr. 2022 Apr 25;129(7):1213–20. doi: 10.1017/S0007114522001209 (PMC10011590; doi:10.1017/S0007114522001209)
Supplement: Supplementary file 1 [file S0007114522001209sup001.docx]

| **Supplementary Table 1. Hazard ratio (HRs) and 95% confidence intervals (CIs) of cardiovascular mortality according to quintiles of vitamin B1 and B3 intakes after exclusion of persons who died within the first 3 years of follow-up** | | | | | | | |  |
| --- | --- | --- | --- | --- | --- | --- | --- | --- |
|  |  |  |  |  |  |  |  |  |
|  |  | Q1 | Q2 | Q3 | Q4 | Q5 | *P* for trend |  |
|  |  | (low) |  |  |  | (high) |  |  |
| Total No. |  | 11569 | 11569 | 11570 | 11569 | 11569 |  |  |
| ***Vitamin B1*** | |  |  |  |  |  |  |  |
| Person-years | | 183738 | 186591 | 192955 | 198478 | 202897 |  |  |
| **CVD** |  |  |  |  |  |  |  |  |
|  | No. of case | 631 | 653 | 608 | 630 | 642 |  |  |
|  | Model 1 | 1.00 | 0.92(0.82-1.03) | 0.85(0.76-0.96) | 0.82(0.73-0.93) | 0.80(0.71-0.90) | <0.001 |  |
|  | Model 2 | 1.00 | 0.98(0.86-1.12) | 0.88(0.76-1.01) | 0.86(0.74-0.99) | 0.88(0.75-1.03) | 0.07 |  |
| **Stroke** |  |  |  |  |  |  |  |  |
|  | No. of case | 272 | 282 | 271 | 293 | 302 |  |  |
|  | Model 1 | 1.00 | 0.92(0.78-1.10) | 0.89(0.75-1.06) | 0.90(0.76-1.07) | 0.89(0.75-1.06) | 0.22 |  |
|  | Model 2 | 1.00 | 1.04(0.85-1.26) | 0.97(0.79-1.20) | 1.00(0.80-1.25) | 1.05(0.82-1.34) | 0.81 |  |
| **Hemorrhagic stroke** | |  |  |  |  |  |  |  |
|  | No. of case | 110 | 98 | 88 | 97 | 110 |  |  |
|  | Model 1 | 1.00 | 0.80(0.60-1.06) | 0.69(0.51-0.93) | 0.72(0.53-0.96) | 0.77(0.58-1.03) | 0.08 |  |
|  | Model 2 | 1.00 | 0.88(0.63-1.21) | 0.75(0.53-1.08) | 0.80(0.55-1.16) | 0.96(0.64-1.42) | 0.97 |  |
| **Ischemic stroke** | |  |  |  |  |  |  |  |
|  | No. of case | 139 | 163 | 156 | 167 | 167 |  |  |
|  | Model 1 | 1.00 | 1.03(0.82-1.29) | 1.01(0.80-1.28) | 1.00(0.79-1.27) | 0.98(0.77-1.24) | 0.80 |  |
|  | Model 2 | 1.00 | 1.15(0.88-1.50) | 1.08(0.81-1.44) | 1.09(0.80-1.48) | 1.12(0.80-1.56) | 0.77 |  |
| **Ischemic heart disease** | |  |  |  |  |  |  |  |
|  | No. of case | 165 | 155 | 130 | 115 | 101 |  |  |
|  | Model 1 | 1.00 | 0.89(0.71-1.12) | 0.76(0.60-0.97) | 0.64(0.50-0.82) | 0.54(0.42-0.70) | <0.001 |  |
|  | Model 2 | 1.00 | 0.90(0.69-1.16) | 0.75(0.56-1.00) | 0.64(0.47-0.88) | 0.57(0.40-0.81) | <0.001 |  |
| **Myocardial Infarction** | |  |  |  |  |  |  |  |
|  | No. of case | 125 | 116 | 97 | 85 | 73 |  |  |
|  | Model 1 | 1.00 | 0.90(0.70-1.17) | 0.78(0.59-1.02) | 0.65(0.48-0.87) | 0.54(0.40-0.73) | <0.001 |  |
|  | Model 2 | 1.00 | 0.90(0.67-1.21) | 0.75(0.54-1.05) | 0.64(0.44-0.92) | 0.56(0.37-0.84) | 0.001 |  |
| **Heart failure** | |  |  |  |  |  |  |  |
|  | No. of case | 84 | 100 | 95 | 105 | 116 |  |  |
|  | Model 1 | 1.00 | 0.91(0.67-1.22) | 0.82(0.60-1.11) | 0.82(0.61-1.11) | 0.86(0.64-1.16) | 0.31 |  |
|  | Model 2 | 1.00 | 0.94(0.67-1.33) | 0.79(0.55-1.15) | 0.77(0.52-1.13) | 0.82(0.54-1.23) | 0.76 |  |
|  |  |  |  |  |  |  |  |  |
| ***Vitamin B3*** | |  |  |  |  |  |  |  |
| Person-years | | 181396 | 188564 | 195301 | 198065 | 201334 |  |  |
| **CVD** |  |  |  |  |  |  |  |  |
|  | No. of case | 671 | 620 | 629 | 602 | 642 |  |  |
|  | Model 1 | 1.00 | 0.89(0.79-0.99) | 0.88(0.78-0.98) | 0.82(0.74-0.92) | 0.84(0.75-0.94) | 0.001 |  |
|  | Model 2 | 1.00 | 0.92(0.82-1.04) | 0.91(0.80-1.02) | 0.85(0.75-0.97) | 0.89(0.78-1.01) | 0.06 |  |
| **Stroke** |  |  |  |  |  |  |  |  |
|  | No. of case | 300 | 257 | 297 | 289 | 277 |  |  |
|  | Model 1 | 1.00 | 0.82(0.70-0.97) | 0.93(0.79-1.10) | 0.89(0.75-1.05) | 0.81(0.69-0.97) | 0.06 |  |
|  | Model 2 | 1.00 | 0.87(0.73-1.05) | 0.98(0.82-1.18) | 0.93(0.77-1.13) | 0.87(0.72-1.07) | 0.18 |  |
| **Hemorrhagic stroke** | |  |  |  |  |  |  |  |
|  | No. of case | 125 | 93 | 97 | 91 | 97 |  |  |
|  | Model 1 | 1.00 | 0.69(0.52-0.90) | 0.69(0.52-0.90) | 0.63(0.47-0.83) | 0.64(0.48-0.84) | 0.002 |  |
|  | Model 2 | 1.00 | 0.71(0.53-0.95) | 0.70(0.52-0.95) | 0.64(0.47-0.88) | 0.67(0.49-0.93) | 0.04 |  |
| **Ischemic stroke** | |  |  |  |  |  |  |  |
|  | No. of case | 154 | 132 | 178 | 172 | 156 |  |  |
|  | Model 1 | 1.00 | 0.83(0.66-1.05) | 1.12(0.90-1.39) | 1.06(0.85-1.33) | 0.93(0.74-1.16) | 0.95 |  |
|  | Model 2 | 1.00 | 0.89(0.70-1.15) | 1.19(0.93-1.52) | 1.12(0.87-1.45) | 1.00(0.76-1.31) | 0.99 |  |
| **Ischemic heart disease** | |  |  |  |  |  |  |  |
|  | No. of case | 166 | 145 | 130 | 108 | 117 |  |  |
|  | Model 1 | 1.00 | 0.89(0.71-1.12) | 0.79(0.63-1.01) | 0.66(0.51-0.84) | 0.68(0.54-0.87) | <0.001 |  |
|  | Model 2 | 1.00 | 0.92(0.72-1.17) | 0.83(0.64-1.08) | 0.70(0.53-0.93) | 0.77(0.58-1.02) | 0.02 |  |
| **Myocardial Infarction** | |  |  |  |  |  |  |  |
|  | No. of case | 126 | 113 | 93 | 81 | 83 |  |  |
|  | Model 1 | 1.00 | 0.93(0.72-1.21) | 0.76(0.58-1.01) | 0.66(0.5-0.88) | 0.65(0.49-0.87) | <0.001 |  |
|  | Model 2 | 1.00 | 0.94(0.71-1.24) | 0.78(0.57-1.05) | 0.69(0.5-0.95) | 0.71(0.51-0.99) | 0.01 |  |
| **Heart failure** | |  |  |  |  |  |  |  |
|  | No. of case | 87 | 104 | 102 | 86 | 121 |  |  |
|  | Model 1 | 1.00 | 1.02(0.77-1.37) | 0.96(0.71-1.28) | 0.77(0.57-1.05) | 1.04(0.78-1.38) | 0.77 |  |
|  | Model 2 | 1.00 | 1.06(0.78-1.46) | 0.98(0.70-1.36) | 0.78(0.55-1.10) | 1.04(0.75-1.46) | 0.54 |  |
| CVD, cardiovascular disease  Model 1: Age- and sex- adjusted hazard ratio (95% CIs) by Cox proportional hazard model. | | | | | |  |  |  |
| Model 2: Adjusted further for histories of hypertension and diabetes, smoking status, body mass index, | | | | | | |  |  |
| hours of walking, hours of sports, educational status, perceived mental stress and ethanol intake, multivitamin supplementation, | | | | | | |  |  |
| quintiles of energy-adjusted sodium, saturated fatty acids, and total energy intake. | | | | | |  |  |  |
|  | | | | | |  |  |  |

| **Supplementary Table 2: The mean values and proportional characteristics in respondents and non-respondents to the food frequency questionnaire** | | | | | | | |
| --- | --- | --- | --- | --- | --- | --- | --- |
|  |  | Respondents |  | Non-respondents |  | *P* for difference |  |
| No. at risk |  | 61787 |  | 24614 |  |  |  |
| Female, % |  | 60.6 |  | 54.0 |  |  |  |
| Age, years |  | 56.5 |  | 61.2 |  | < 0.001 |  |
| Body mass index, kg/m^2^ |  | 22.8 |  | 22.8 |  | 0.66 |  |
| Ethanol intake, g/d |  | 28.2 |  | 29.1 |  | 0.003 |  |
| Current smoker, % |  | 24.6 |  | 30.5 |  | <0.001 |  |
| History of hypertension, % |  | 21.3 |  | 28.0 |  | <0.001 |  |
| History of diabetes, % |  | 4.9 |  | 7.6 |  | <0.001 |  |
| Sports ≥5 h/wk, % |  | 5.4 |  | 6.4 |  | <0.001 |  |
| Walking ≥5 h/wk, % |  | 50.3 |  | 48.9 |  | 0.001 |  |
| > 18 y education, % |  | 13.8 |  | 12.1 |  | <0.001 |  |
| High mental stress, % |  | 22.3 |  | 18.2 |  | <0.001 |  |

**Supplement figure 1: Participants’ flow chart**

110,585 participants

(46,395 men and 64,190 women)

86,401 participants

13 areas where a common FFQ was not use

n=24,184

61,787 participants

58,645 participants

(23,099 men and 35,546 women)

Exclusion of non-respondents to FFQ

n=24,614

Those with history of CVD or cancer

n=3,142

58,302 participants

(22,989 men and 35,313 women)

Those who had extreme energy intakes:

n=343

Supplementary methods

The hypothesized confounding factors included age, sex, medical history of hypertension and diabetes, smoking status, ethanol intake, hours of sports, hours of walking, quintiles of body mass index (BMI) in kg/m^2^, years of education, perceived mental stress, daily utilization of multivitamin supplementation, energy-adjusted quintiles of sodium and saturated fatty acids intakes, and total energy intakes. For the medical history of hypertension and diabetes, the participants were asked, ‘Do you have any of the following diseases?’ Hypertension, and diabetes were included in the list, among many other diseases. Participants could choose one option of the following, “no”, “yes, under treatment”, “yes, already treated”, and “yes, not treated at all”. We categorized the participants in the model as (yes, no, or missing). For smoking status, there were two questions ‘Do you currently smoke?’ With options ‘never’, ‘ex-smoker, and ‘current smoker’. The second question was ‘How many cigarettes do you smoke per day if you are a current smoker?’ With open answers for the number of cigarettes. We categorized the participants in the model as (never, ex-smoker, current smoker of 1-19 cigarettes/day, current smoker of more than 20 cigarettes/day, or missing). For drinking status, participants were first asked ‘Do you currently drink alcohol?’ With options ‘never’, ‘I quit drinking, and ‘yes’. For those who answered ‘yes, there were two further questions ‘What kind of alcohol do you drink? Beer, Japanese sake, Whiskey, spirits, wine, or others? And ‘How much do you drink?’ With options for frequency of drinking (nearly every day; three or four times a week; once or twice a week; and averagely less than once a week). We categorized the participants in the model as (never, ex-drinker, current drinker of 0.1-22.9 g ethanol/day, 23.0-45.9 g ethanol/day, 46.0-68.9 g ethanol/day, more than 69.0 g ethanol/day, or missing). For hours of sports, participants were asked ‘How many hours do you practice sports a week?’ With options ‘almost never’, ‘1 to 2 hours a week’, ‘3 to 4 hours a week’, or ‘over 5 hours a week’. We categorized the participants in the model as (almost never, 1-2 hours/week, 3-4 hours/week, more than 5 hours/week, or missing). Also, there was one question about walking ‘How many minutes do you walk a day?’ With options ‘almost never’, ’0 to 30 minutes’, ’30 to 60 minutes, or ‘over 60 minutes. We categorized the participants in the model as (almost never, 0.5 hours/day, 0.6-0.9 hours/day, 1 hour or more/day, or missing). For years of education, participants gave a number to this question ‘How many years of education did you receive?’ We categorized the participants in the model as (≤15 years, 16–18 years, >18 years, or missing). There was one question for perceived mental stress: ‘Do you feel stress in your daily life?’ With options ‘so much’, ‘much’, ‘normal’, or ‘less’. We categorized the participants in the model as (low, normal, high, or missing). For daily utilization of multivitamin supplementation, participants were asked ‘Do you use multivitamin supplementation daily?’ With options ‘yes’ or ‘no’. We categorized the participants in the model as (yes, no or missing).
